# Siglec-15 Silencing Inhibits Cell Proliferation and Promotes Cell Apoptosis by Inhibiting STAT1/STAT3 Signaling in Anaplastic Thyroid Carcinoma

**DOI:** 10.1155/2022/1606404

**Published:** 2022-06-20

**Authors:** Xiaofeng Hou, Chao Chen, Xiaodong He, Xiabin Lan

**Affiliations:** ^1^Department of General Surgery, The Second Clinical Medical College, Lanzhou University, Lanzhou, 730030 Gansu, China; ^2^Department of Head & Neck Oncology Surgery, The Cancer Hospital of the University of Chinese Academy of Sciences (Zhejiang Cancer Hospital), Institute of Basic Medicine and Cancer (IBMC), Chinese Academy of Sciences, Hangzhou, 310022 Zhejiang, China; ^3^Key Laboratory of Head & Neck Cancer Translational Research of Zhejiang Province, Hangzhou, 310022 Zhejiang, China

## Abstract

Thyroid cancer (THCA) represents a frequently seen endocrine cancer, which can be divided as anaplastic thyroid carcinoma (ATC), follicular thyroid carcinoma (FTC), and papillary thyroid carcinoma (PTC). A total of 362 IDEGs were obtained from TCGA-THCA and IMMPORT databases, which were found to be related to BP, CC, MF, and STAT signaling pathway upon GO functional annotation and KEGG analysis. This work identified 23 survival-related hub genes using WGCNA and uniCOX analysis. In addition, a risk prognosis model was constructed to obtain a signature involving fifteen IDEGs. According to survival and univariate along with multivariate analysis, high-risk patients had markedly dismal prognostic outcome compared with low-risk counterparts. Siglec-15 belongs to one of the fifteen IDEG signature, but the precise biological roles in diverse THCA subtypes are largely unclear. In this work, Siglec-15 expression evidently increased in ATC and FTC samples compared with matched surrounding PTC and THCA samples, which was used as a diagnostic biomarker for THCA. Siglec-15 RNAi significantly inhibited cell proliferation and promoted cell apoptosis. Meanwhile, Siglec-15 knockout suppressed the expression of STAT1, STAT3, and VEGF and promoted that of cleaved caspase-3. In *vivo* experiments revealed that transfection with vectors expressing STAT1 and STAT3 inhibited the Siglec-15 RNAi-induced inhibition on tumor growth and the increases in CD4^+^/CD8^+^ ratio. In conclusion, Siglec-15 expression increases in ATC and FTC, which promotes THCA occurrence via the STAT1/STAT3 signaling, in particular for FTC and ATC. Therefore, it is the possible marker that can be used to diagnose and treat THCA.

## 1. Introduction

Thyroid cancer (THCA) accounts for a frequently occurring human malignancy, its incidence shows an increasing trend globally, and THCA is expected to be the fourth largest cancer type globally over the past 50 years [1]. The increasing incidence of THCA can be attributed to several factors below: (1) the increased detection rate of early tumors, (2) increased personal risk factors (e.g., obesity), (3) increased environmental risk factors (e.g., radiation), and (4) the improved diagnostic techniques related to the enhanced personal health awareness (e.g., magnetic resonance imaging, MRI) [2]. However, data from the past decade show that THCA exhibits an increasing mortality rate as the incidence of advanced THCA (including large tumors and locally advanced or metastatic tumors) increases every year [3]. More than 90% of the THCA subtypes are well-differentiated; among which, papillary thyroid carcinoma (PTC) shows the highest morbidity, occupying over 80% of the THCA cases, whereas follicular thyroid carcinoma (FTC) and anaplastic thyroid carcinoma (ATC) rank the second and third places, respectively [4, 5]. These differentiated THCAs are treated with traditional interventions, but the problems of poor prognosis and insensitivity to radiotherapy are encountered. Therefore, immunotherapy is currently considered as the fourth cancer treatment after surgery, chemotherapy, and radiotherapy [6]. Immune response is critical in cancer treatment. It has long been considered to enhance the efficacy or the extent of antitumor immune responses by enhancing the immune activation mechanisms, ultimately killing the target tumor. Traditional treatments have been demonstrated to be effective on some cancers [7]. But in most cases, they cannot attain expected therapeutic effect. Due to the difference in tumor microenvironment (TME), the above strategies can probably extensively activate the both immunity, which can significantly increase the immune-associated side effect rate or even result in autoimmune disorders [8]. However, tumors have developed the immune escape mechanism by which tumors actively utilize a variety of pathways for delaying, altering, or even blocking the anticancer immunity. In this way, it blocks the immune system's ability to effectively suppress cancer development, finally inducing progressive disease (PD). Full-body activation of the immune system or even an increase in peripheral tumor-specific T cells may not result in tumor regression [9]. Therefore, some scholars believe that tumor immunotherapy is important not only to strengthen the immune system but also to restore the function of the tumor immune microenvironment (TIME). The normal human body functioning relies on diverse balanced and stable systems like immune system [9].

Immunotherapy makes use of a patient's own immune system to produce an immune response to kill tumor cells in the body, thereby resulting in persistent remission [10]. Currently, more immunotherapies are used in the clinical settings to reverse T cell tolerance and reestablish the efficient anticancer immunoreaction called immune checkpoint (ICP) inhibition, which are achieved by blocking the inhibitory interactions between tumor-infiltrating T cells (TIICs) and tumor cells. Immune checkpoint inhibitors (ICIs), containing anti-PD-1, anti-PD-L1, and anti-CTLA-4, can escape ICPs to restore and enhance functions of antitumor T cells and attain good clinical results [11]. The anti-PD-1/PD-L1 treatment, which is first used in the treatment of melanoma and blood cancers [12], represents a widely recognized and highly efficient tumor immunotherapy. But it is effective on only 20-30% of solid human tumors and only 20% of head and neck squamous cell carcinomas (HNSCCs) [13]. Recently, PD-L1 expression can be detected within only about 50% of PTCs [14]. In these cases, PD-1 and PD-L1 are poorly treated. This low efficiency also suggests that there may be other potential immune suppression pathways. Therefore, the search for novel ICPs will add to the therapeutic spectrum of cancer immunotherapy.

Thanks to the development of a public web-based bioinformation service platform and the rapid development of bioinformatics, an increasing number of studies have employed bioinformatics analysis to mine tumor immune-related molecules in recent years. In the study reported by Wang et al., after several rounds of screening and validation of a high-throughput whole-genome T cell activity array (TCAA) system, Siglec-15 was detected as an inhibitory candidate [15]. According to TCGA database analysis, the mRNA expression of Siglec-15 increased within diverse cancers, such as colonic, thyroid, endometrioid, liver, lung, kidney, and bladder carcinomas [15, 16]. Siglecs belong to the sialic acid- (SA-) bound immunoglobulin- (Ig-) like lectin family, which contribute to the interactions between cells or between cells and pathogens through the identification of SA-containing glycan chains [17]. Therefore, they have critical effects on regulating both congenital and acquired immunity. NC318 is the experimental monoclonal antibody (mAb) of Siglec-15. Dr. Anthony Tolcher reported the preliminary findings from the Phase I study of NC318, which were that among the 49 patients with a variety of tumor types, containing non-small-cell lung carcinoma (NSCLC), NC318 was safe and well tolerated, and primary adverse reactions, including diarrhea and elevated levels of asymptomatic amylase and lipase, occurred mainly in cases showing decreased PD-L1 levels [18]. Recent studies have reported encouraging results for specific anti-Siglec-15 mAbs (*α*-S15) from different mouse models of tumors, and the Phase I clinical trials of humanized anti-Siglec-15 mAb (NC318) for solid tumors (NCT03665285) are ongoing [19]. This suggests that Siglec-15 is a key gene in tumor immunotherapy.

As revealed by the survival analysis based on TCGA database and THCA clinical data, Siglec-15 upregulation was related to overall survival (OS). Typically, Siglec-15 can partially account for the reason regarding the low (20-30%) efficiency of anti-PD-1/PD-L1 treatment in human solid tumors [20]. Notably, PD-1/PD-L1 pathway stands for a mechanism of tumor immune escape. Anti-Siglec-15 is the possible treatment option in PD-1/PD-L1 treatment-insensitive patients, which is also the important anti-PD-1/PD-L1 complement.

## 2. Materials and Methods

### 2.1. TCGA Data

This work acquired transcriptome data of the THCA cohort (including 510 tumor samples and 58 normal adjacent tissues, clinical data of THCA cases) in TCGA-THCA project (http://portal.gdc.cancer.gov/).

### 2.2. Identification of Differentially Expressed Genes (DEGs)

DEGs between THCA samples and normal tissues were identified by R package “limma” function (version 4.1.2) upon the thresholds of false discovery rate (FDR) < 0.05 and |log2 fold change (log2FC| > 1). Later, consensus DEGs were identified between 2 groups.

### 2.3. Acquisition of Immune-Related Genes (IRGs)

This work obtained IRGs in IMMPORT database (https://www.immport.org/home) and later discovered consensus DEGs between 2 groups. Afterwards, a Venn plot was drawn to display the results. Clustering analysis of these DEGs was conducted using heat map in R.

### 2.4. GO Functional Annotation and KEGG Pathway Enrichment Analysis

For better exploring DEGs' biological functions, the R package clusterprofiler function was employed for data analysis and visualization of the enriched functional terms and pathways. The valuable data were acquired in the above analyses, with *p* < 0.05 indicating significant enrichment.

### 2.5. Coexpression Network Construction and Module Functional Analysis

Firstly, this work analyzed the expression profiling patterns of immune-related DEGs (IDEGs) to examine the suitability of genes and samples. Then, the R software “WGCNA” package was adopted for establishing a coexpression network based on those IMDEGs. After functioning of pairwise genes by Pearson's correlation matrices, this work established the weighted adjacent matrix using the power function amn = |cmn|*β* (where amn denotes the adjacent of gene m to gene n, while cmn represents Pearson's correlation between genes m and n). Subsequently, this work adopted the soft-thresholding *β* parameter for emphasizing the potent gene associations and for penalizing the weak associations. Later, the adjacency matrix was converted into the topological overlap matrix (TOM) for measuring a gene network connectivity (total adjacent of this gene with the remaining genes) to generate a network. The TOM-based dissimilarity measure was adopted for mean linkage hierarchical clustering analysis to build a gene dendrogram (minimal size (gene group) =50); as a result, genes were classified to same gene module with close expression patterns. Moreover, module eigengenes' dissimilarity was also determined. For identifying tumor-related modules, the above gene modules were subject to functional enrichment.

### 2.6. Prognostic Model Construction Based on DEGs

This work enrolled a total of 510 THCA samples to analyze the clinicopathological features and prognostic outcome. Later, prognostic DEGs were identified by univariate Cox regression. Risk score of genes was determined by the following formula: gene level 1∗genecoef1 + gene level2∗genecoef 2 + gene level3∗genecoef 3 + ⋯+gene level N∗genecoef N. This work adopted “survminer” and “survival” functions of R software to analyze the best threshold by log-rank test (two-sided). All cases were categorized as low- or high-risk group based on the as-determined threshold. By adopting “survivalROC” function of R software, this work plotted time-dependent receiver operating characteristic (t-ROC) curves for assessing whether our constructed prognostic model was significant in prognosis prediction. Moreover, the log-rank test and Kaplan-Meier (KM) approach were utilized for comparing difference in survival between the low- and high-risk groups with “survival” function in R software. Later, we validated the prognostic model's significance in prognosis prediction using the test and the entire cohorts. Thereafter, univariate as well as multivariate Cox regression was carried out for analyzing factors independently predicting prognosis of THCA, and forest plots were drawn for result visualization.

### 2.7. Evaluation of Clinicopathological Characteristics Correlated with the Immune Subtypes

This work examined gene profiling patterns of 500 TCGA-THCA samples based on 15 genes related to clinicopathological characteristics and prognostic outcome of THCA cases. Later, we classified all cases as 2 groups in line with the risk score and analyzed THCA samples based on the relations of diverse variable combinations with immune subtypes by adopting RColorBrewer and nonnegative matrix factorization (NMF) functions in R software.

### 2.8. Tissue Specimens

This work acquired a total of 86 THCA tissues (tumor group) and corresponding normal tissues (control group) in the First Affiliated Hospital of Lanzhou University from 2005 to 2010.  Table 1 displays the tumor clinicopathological features. All cases were treatment-naive and provided the informed consent preoperatively. Two experienced pathologists from the Pathology Department of our institution were responsible for the histological examination of all tissues following the World Health Organization (WHO) criteria. Our study protocols gained approval from Institutional Review Board and Human Ethics Committee of our institution, and this work was performed following the Declaration of Helsinki. With regard to histological subtypes of those collected samples, 31 were ATC, 26 were PTC,16 were FTC, and 13 were thyroid adenomas.

### 2.9. Cell Culture and Treatment

This work obtained FRO (undifferentiated ATC cells), ARO (undifferentiated ATC cells), and WRO (poorly differentiated THCA cells) cell lines from Cell Bank of Chinese Academy of Sciences as a gift and kept them within DMEM (Invitrogen, Carlsbad, CA) that contained 1% penicillin-streptomycin (Invitrogen, Carlsbad, CA) as well as 10% FBS (HyClone, Logan, UT).

FRO, ARO, or WRO (2 × 10^3^/100 *μ*l) cells were cultured within the 96-well plates that contained agonistic anti-CD40 monoclonal antibody (mAb) (5C11) at diverse doses (10.0 *μ*g/ml)for 24 h.

### 2.10. Siglec-15 Knockdown, STAT1 and STAT3 Overexpression, and Cell Transfection

To deplete siglec-15 expression, this study inserted human shRNA sequences in pSuper-retro-puro plasmid for generating the pSuper-retro-siglec-15-RNAi(s) (Genepharma, Bioscience, Shanghai, China). Thereafter, retrovirus vector was produced and transfected into cells according to the previous description [21]; after transfection for a 48-h period, 0.5 *μ*g/ml puromycin was further added to treat cells for a 10-14-day period, so as to select the stable cell lines.

Sangon (Shanghai, China) was responsible for preparing STAT1/STAT3 cDNA-expressing pIRSE2 vectors, along with empty pIRSE2 vectors. Thereafter, when THCA cells reaching 70–80% density, they were harvested and transfected with 50 nM vectors for a 5 h period by adopting Lipofectamine 2000 reagent (Thermo Fisher Scientific). The cells were later incubated with freshly prepared medium prior to later analysis.

### 2.11. Clone Forming Assay

In clone forming assay, we digested THCA cells and inoculated them into the 6-well plates at 5 × 102/well that were filled with 1%penicillin-streptomycin and 10% FBS under 37°C and 5% CO_2_ conditions. The original medium was discarded after 2 weeks, followed by PBS rinsing of cells thrice. Later, anhydrous ethanol was utilized to fix cells for a 30 min period, followed by 20 min staining using hematoxylin. The number of colonies that contained at least 50 cells was counted.

### 2.12. Flow Cytometry (FCM)

Rat anti-mouse mAbs (Affymetrix eBioscience), which included fluorescein isothiocyanate- (FITC-) labeled anti-CD4 (0.3 *μ*l, No11-0041) as well as phycoerythrin- (PE-) labeled anti-CD8a (0.7 *μ*l, No11-0081), were utilized to stain cells from blood specimens for a 15 min period in dark. After adding hemolysin (250 *μ*l), the cells were subject to further 15 min incubation in the dark and PBS rinsing thrice. CD4^+^/CD8^+^ ratio was determined as the ratio of average fluorescence intensity of CD4^+^ lymphocytes to that of CD8^+^ cells detected using the flow cytometer (Beckman coulter, Navios, USA).

THCA cells undergo certain treatments, including staining using Annexin V-FITC and propidium iodide (PI) Apoptosis Detection Kit I (BD, USA) in line with specific instructions, and cell apoptosis was analyzed through FACS (BD, USA).

Data analysis was completed using Cell Quest Research Software (BD, USA).

### 2.13. Animal Studies

This work obtained the six- to eight-week-old female C57BL/6 mice in SLAC Laboratory Animal Co., Ltd., and randomized them as 3 groups (*n* = 3 each). To establish the tumor xenograft models, 6 × 10^6^ siglec-15 RNAi-transfected cells, vector-expressing STAT1-transfected cells, and control cells were subcutaneously injected in each nude mouse via right armpit. Tumor size was examined at 3 days after injection at 2-day intervals. Thereafter, tumor volume was decided with the formula below (length × width^2^ × 0.5). Peripheral blood samples were obtained following 20-day oral feeding; at 35 days later, each mouse was euthanized to dissect tumor tissues. Afterwards, tumor tissues in each mouse were subject to paraffin embedding and slicing into 5 *μ*m sections prior to analysis. Our study protocols gained approval from the Laboratory Animal Center of Lanzhou University. Each animal experiment was performed following institution guidelines.

### 2.14. Immunohistochemical Analysis (IHC)

In IHC assay, both human and mouse paraffin-embedded THCA tissues were utilized. Each patient provided the informed consent for clinical sample use, and the study protocols were approved by the Institutional Research Ethics Committee. Each section was subject to immunostaining with anti-siglec-15, anti-Ki67, and anti-VEGF antibodies (Beyotime, Shanghai, China). Later, the AxioVision Rel.4.6 computerized image analysis system (Carl Zeiss, Oberkochen, Germany) was employed for capturing immunostaining images in line with the previous description. Then, two reviewers were responsible for reviewing and scoring the immunostaining degree of formalin-fixed, paraffin-embedded (FFPE) sections according to positively stained tumor cell percentage along with staining intensity.

### 2.15. RNA Extraction, cDNA Preparation, and Quantitative Reverse Transcription PCR (qRT-PCR)

The total RNA was extracted by TRIzol (Thermo Fisher Scientific, USA). The extracted RNA purity was determined by the NanoDrop-1000 spectrometer (NanoDrop, Thermo Fisher Scientific, Waltham, Massachusetts, USA). The first-strand cDNA was synthesized by the high-capacity cDNA Reverse Transcription kit (Applied Biosystems, USA) according to the manufacturer's instructions. Subsequently, qRT-PCR was performed on a ABI 7500 Real-Time PCR system (Applied Biosystems, USA) using the ChamQTM Universal SYBR® qPCR Master Mix (Vazyme, China) under the following cycling conditions: 10 min under 95°C, 1 min under 95°C, 1 min under 53°C, and 1 min under 72°C for 40 cycles, followed by 5 min under 72°C. Sequences of primers utilized in this work were siglec-15 (F) 5′-GTTCTCGGGCACCTTGG-3′ and (R) 5′-AGCTCCGAAATGGTTGTCC-3′ and GAPDH (F) 5′-GGGGCTCTCCAGAACATC-3′ and (R) 5′-TGACACGTTGGCAGTGG-3′. Target gene expression was normalized to GAPDH and calculated by 2^–*ΔΔ*Ct^ approach. Every assay was conducted thrice independently.

### 2.16. Western Blot (WB) Analysis

The radioimmunoprecipitation assay buffer (RIPA, Beyotime, China) was employed for extracting total cellular proteins in THCA cells after transfection. WB assay was carried out to measure protein levels according to previous description. This work acquired primary antibodies against siglec-15 (1 : 500; Abcam), VEGF (1 : 800; Abcam), STAT1 (1 : 500; Beyotime), STAT3 (1 : 500; Beyotime), GAPDH (1 : 500; Beyotime), and cleaved caspase-3 (1 : 500; Beyotime), together with relevant secondary antibody (1 : 500) in Santa Cruz Biotechnology (Shanghai, China), with GAPDH being the endogenous reference. Odyssey was employed for band detection, whereas Image Studio Software (LI-COR Bioscience, Lincoln, Nebraska, USA) was utilized in band analysis.

### 2.17. Statistical Analysis

SPSS20.0 (IBM, Chicago, IL, USA) was utilized for statistical analyses. Significant differences were compared between the 2 groups by paired Student's *t*-test (two-tailed). The Kaplan-Meier (KM) approach was utilized to assess OS of THCA cases, and distribution of survival between the 2 groups was analyzed by log-rank test. Relations of siglec-15 level with pathological features were analyzed by chi-square test. Data were displayed as mean ± SD of 3 separate assays. *p* < 0.05 stood for statistical significance.

## 3. Results

### 3.1. Identification of THCA-Related Immune Genes (TCIGs)

**Figure 1:**
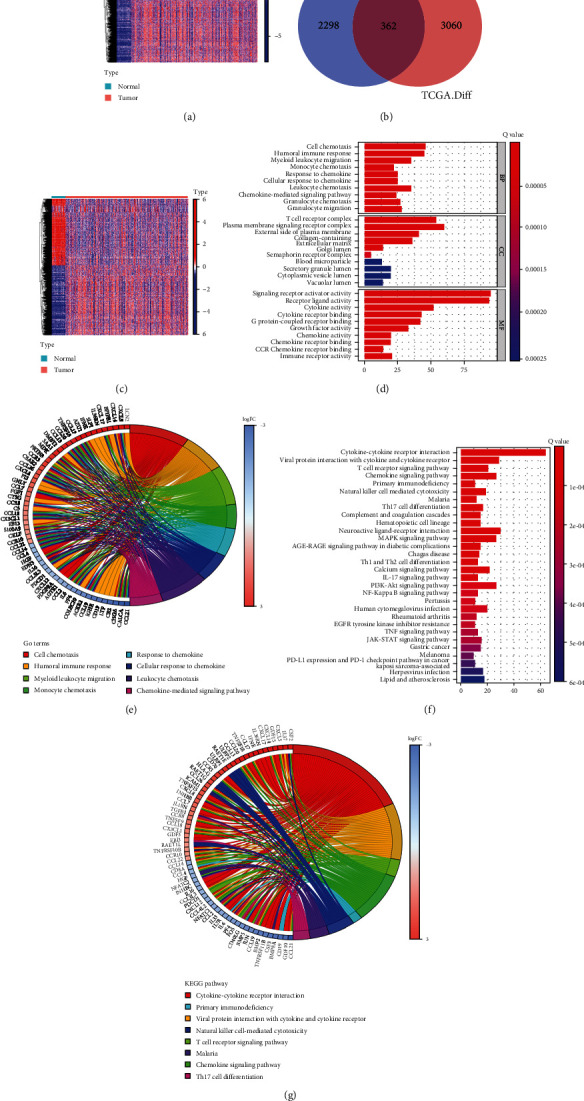
Identification of THCA-related immune genes. (a) Heat map of DEGs in normal and tumor samples in TCGA-THCA dataset. (b) Venn plots showed immune-related DEGs. (c) Heat map of IDEGs in normal and tumor samples in TCGA-THCA dataset. (d) The top 10 enriched GO terms (BP, CC, and MF). (e) Chord plot depicting the relationship between genes and GO terms of BP, CC, and MF. (f) The top 30 enriched KEGG pathways. (g) Chord plot depicting the relationship between genes and KEGG pathways.

Based on the TCGA-THCA expression profiles, 3422 DEGs were acquired (containing 1748 upregulated and 1674 downregulated ones) (Figure 1(a)). Meanwhile, 2260 immune genes were obtained from IMMPORT database. Then, altogether, 362 intersected genes obtained by Venn analysis were identified as the TCIGs (Figure 1(b)). The expression patterns of 362 TCIGs are shown in Figure 1(c). Moreover, these 362 TCIGs were further subject to GO as well as KEGG analysis. As a result, the above TCIGs showed close relation to some biological processes (BPs) of GO terms, such as cell chemotaxis, humoral immune response, and response to chemokine; several cellular components (CCs) of GO terms such as T cell receptor complex, blood microparticle, and plasma membrane signaling receptor complex; and several molecular functions (MFs) of GO terms such as growth factor activity and receptor ligand activity, together with signaling receptor activator activity (Figures 1(d) and 1(e)). As for KEGG pathway analysis, the TCIGs were mainly enriched into PD-L1 expression, primary immunodeficiency, PD-1 checkpoint pathway, and JAK-STAT pathway (Figures 1(f) and 1(g)).

### 3.2. WGCNA and Identification of Hub Modules

**Figure 2:**
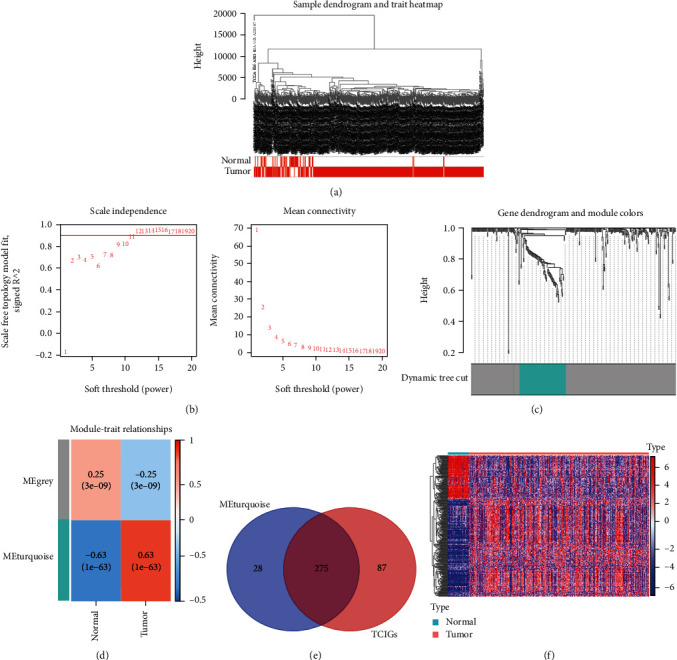
WGCNA and identification of the key module. (a) Hierarchical clustering analysis of TCGA-THCA samples. (b) Determine the best soft threshold using network topology analysis. When *β* = 11, it satisfies the scale-free topology threshold of 0.85. (c) The cluster dendrogram of genes in TCGA. Each branch in the figure represents one gene, and every color below represents one coexpression module. (d) Clustering dendrogram of 510 THCA patients, where 362 IDEGs were clustered based on the dissimilarity measure (1-TOM) and divided into 2 modules. (e) Venn plots showed 275 hub TCIGs. (f) Heat map of TCIGs in normal and tumor samples in TCGA-THCA dataset.

For identifying hub modules closely related to THCA, WGCNA was carried out using TCGA-THCA dataset (Figure 2(a)). Finally, 2 modules were obtained with the cut height and soft-thresholding power of 0.25 (Figure 2(b)) and 11 (scale-free *R*^2^ = 0.85), separately (Figure 2(c); nonclustered DEGs are displayed in gray). Moreover, this work determined turquoise module to be closely related to tumor group based on heat map regarding module–trait relations (Figure 2(d)). Then, a total of 275 intersected genes obtained from Venn analysis were identified as hub TCIGs (Figure 2(e)). Expression of these overlapping genes in THCA based on TCGA-THCA dataset is shown in Figure 2(f).

### 3.3. Identification of OS-Related IRGs and Construction of the Prognostic Model

**Figure 3:**
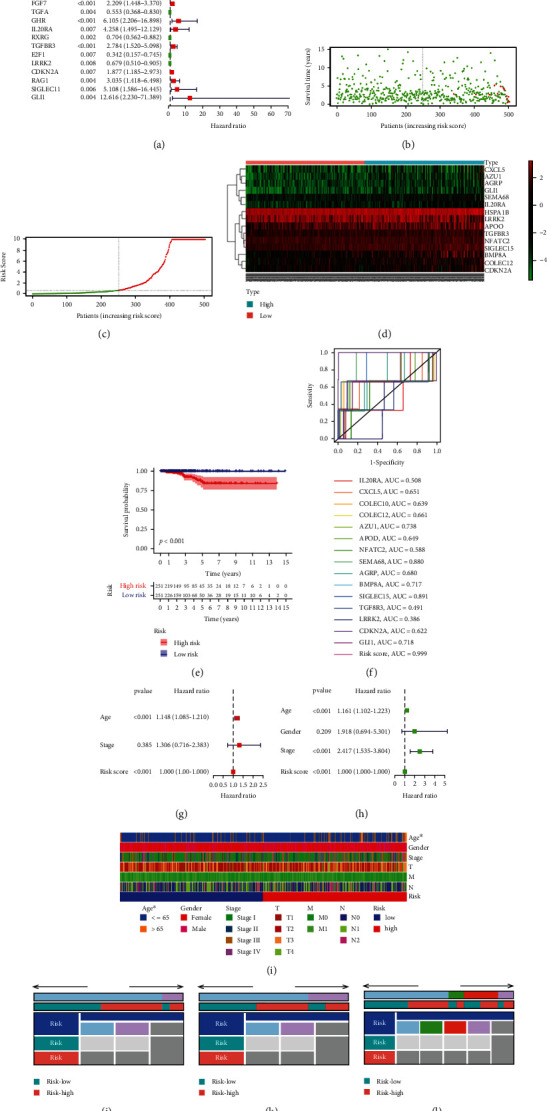
Identification of overall survival-related immune-related genes and construction of the prognostic model. (a) Forest plots of the 23 OS-related hub TCIGs identified by Cox proportional hazard regression. The represented prognostic hub TCIGs with hazard ratios >1 are shown as red dots, and the represented prognostic hub TCIGs with hazard ratios <1 are shown as green dots. (b) Risk score distribution of THCA patients. (c) Survival status of THCA patients with increasing risk score. (d) Heat map of 15 hub TCIGs by definite multivariate Cox regression analysis in the high-risk group and low-risk group. (e) The Kaplan-Meier survival curve analysis for the high- and low-risk group based on the risk score. (f) ROC curve for predicting prognosis gene performance based on risk score. (g) Univariate and (h) multivariate Cox regression analyses of the risk score. (i) Heat map showing the distribution of clinical features between the high- and low-risk groups. The risk scores in different (j) age, (k) gender, and (l) stage for THCA patients. *p* < 0.05.

These 275 hub TCIGs were performed univariate Cox regression analysis, and 23 hub TCIGs significantly correlated with OS were found (Figure 3(a)). Thereafter, these 23 genes were incorporated into multivariate Cox regression, which identified 15 hub TCIGs as the independent prognostic factors (Table 2). For assessing whether these 15 hub TCIGs were of prognostic significance, this work drew risk score plots, heat map, and K-M and ROC curves. As revealed by risk score plots, THCA cases showing large risk score had reduced OS time (Figures 3(b) and 3(c)). Heat map displayed the levels of 15 hub TCIGs among the low- and high-risk cases (Figure 3(d)). As revealed by K-M curve, high-risk THCA cases had reduced OS time (*p* < 0.001, Figure 3(e)). Moreover, ROC curves were drawn based on risk score, and AUC values were determined to be over 0.75, which indicated that our constructed 15-TCIGs prognostic model was highly specific and sensitive. The AUC values for the SEMA6B-ROC curve and the SIGLEC15-ROC curve of the risk scores were 0.88 and 0.891, respectively. In addition, compared with the single gene, our 15-hub TCIG-based prognostic model had the greatest AUC value (Figure 3(f)). Upon univariate as well as multivariate Cox regression, the HRs of OS were 1 (*p* < 0.001, Figures 3(g) and 3(h)). Based on the above findings, our 15-hub TCIG-based prognostic model might be adopted for predicting OS for THCA cases. Afterwards, this work determined the association of risk score for THCA cases with clinical features. The heat map exhibited age, sex, and TNM stage distributions between the 2 groups; among which, difference in age was significant (Figure 3(i)). According to these results, age was significantly different between the 2 groups (Figure 3(j); *p* = 0.012). However, there was no significant difference in gender or stage between the 2 groups (Figures 3(k) and 3(l)).

### 3.4. Siglec-15 Expression Is Upregulated in Different Subtypes of THCA

**Figure 4:**
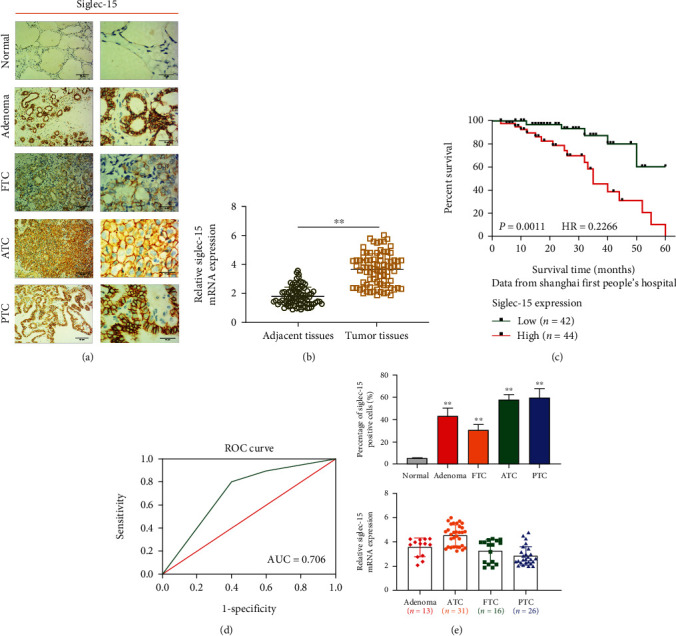
Siglec-15 expression is upregulated in the different subtypes of thyroid cancer. (a) Representative images of Siglec-15 immunostaining in normal thyroid tissue, thyroid adenoma, and thyroid carcinoma specimens with different pathological characteristics. (b) RT-PCR analysis of Siglec-15 expression in adjacent tissues and tumor tissues. (c) The survival curves for low- and high-Siglec-15 expression groups (*p* = 0.0011). (d) The ROC curve validation of the prognostic value of Siglec-15. (e) qRT-PCR analysis of Siglec-15 expression in normal thyroid tissue, thyroid adenoma, and thyroid carcinoma specimens with different pathological characteristics.

For determining siglec-15 protein expression within diverse THCA subtypes, IHC staining was performed in the collected FFPM THCA samples, which included 13 thyroid adenoma, 16 FTC, 26 PTC, and 31 ATC samples. As shown in Figure 4(a), siglec-15 expression could be measured within 55% PTC, 52% ATC, 40.5% thyroid adenoma, and 23% FTC samples. Based on the above findings, siglec-15 level markedly increased within PTC and ATC relative to FTC, thyroid adenoma, and healthy thyroid samples. As a result, siglec-15 is a possible biomarker used to distinguish poorly differentiated THCA from the well-differentiated one. Next, the results of RT-PCR assay showed that siglec-15 expression was universally higher in these 86 THCA tumor samples (Figure 4(b)). The median was used as a cutoff point to classify these 86 THCA patients in two groups, namely, high-siglec-15 and low-siglec-15 expression groups. As a result, THCA patients with higher siglec-15 expression had a poor prognostic outcome (Figure 4(c)). Furthermore, this work plotted ROC curves to analyze the diagnostic value of siglec-15 in THCA cases. The AUC value was determined to be 0.706 (Figure 4(d)), suggesting that Siglec-15 might be the biomarker utilized to diagnose THCA. Thereafter, siglec-15 expression was found to be mainly enriched in ATC samples (Figure 4(e)). Given the findings presented above, this work further analyzed the association of siglec-15 with THCA progression and/or patient prognosis. According to our results, siglec-15 level was markedly associated with THCA subtypes (*p* = 0.001), distant metastasis (*p* < 0.001), and tumor size (*p* = 0.031; Table 1).

### 3.5. Siglec-15 Knockdown Suppresses THCA Cell Growth and Enhances their Apoptosis

**Figure 5:**
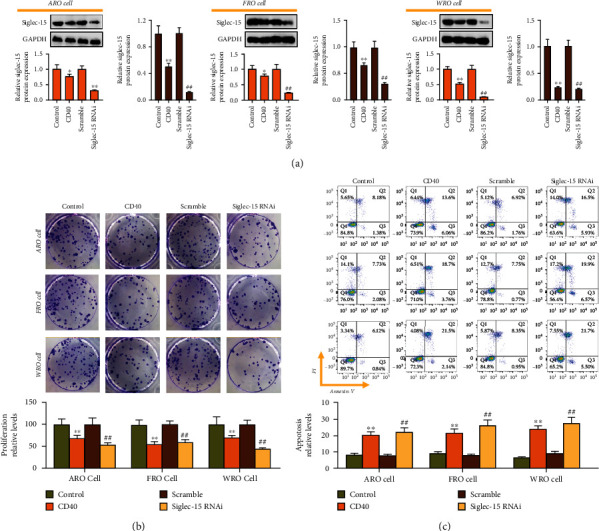
Siglec-15 silencing inhibits the proliferation and promotes its apoptosis in THCA cell lines. (a) Western blot analysis of Siglec-15 expression in THCA cell lines. Clone formation analysis and flow cytometry analysis of cell proliferation (b) and cell apoptosis (c) in THCA cell treatment with agonistic anti-CD40 mAb or Siglec-15 RNAi. ^∗∗^*p* < 0.01 and ^##^*p* < 0.01 indicated statistical significance compared with control group or scramble group.

Firstly, THCA cells were treated with agonistic anti-CD40 mAb and transfected with siglec-15 RNAi (5C11). According to WB assay, transfection with siglec-15 RNAi inhibited siglec-15 expression (Figure 5(a)). Similarly, both treatment with agonistic anti-CD40 mAb and transfection with siglec-15 RNAi inhibited THCA cell proliferation (Figure 5(b)) and promoted their apoptosis (Figure 5(c)). But the inhibition on cell growth induced by siglec-15 knockdown and the promoting effects of siglec-15 silencing on cell apoptosis both were superior to those under 5C11 treatment.

### 3.6. Siglec-15 Silencing Inhibits the Activation of STAT1/STAT3 Signaling Pathway

**Figure 6:**
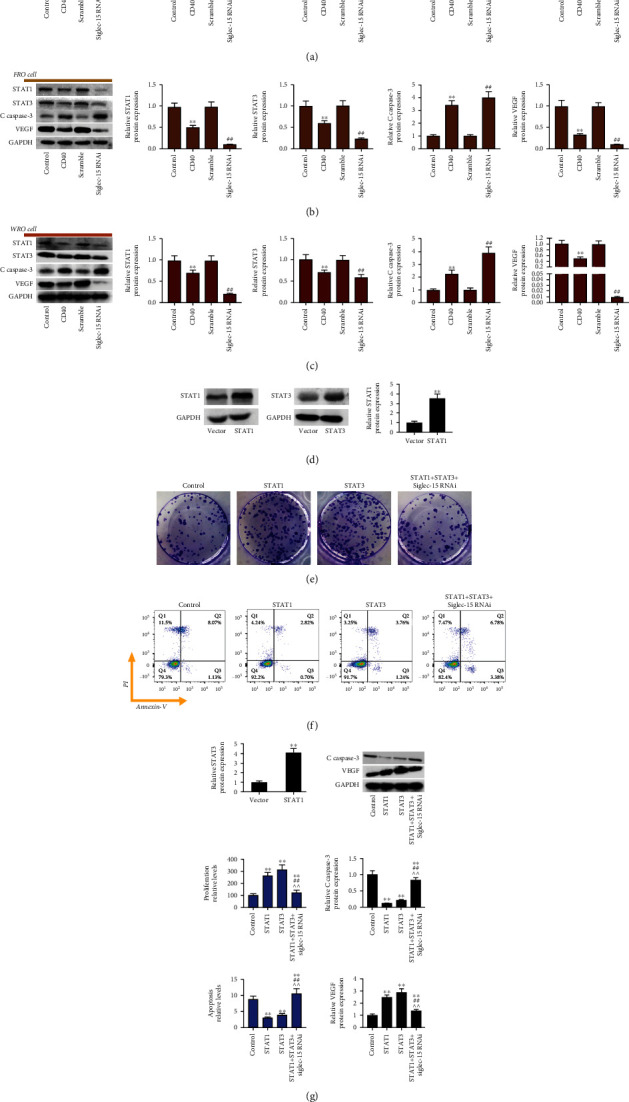
Siglec-15 silencing inhibits the activation of STAT1/STAT3 signaling pathway. Western blot analysis of STAT1 expression, STAT3 expression, cleaved caspase-3 expression, VEGF expression in ARO cells (a), FRO cells (b), and WRO cells (c) in THCA cell treatment with agonistic anti-CD40 mAb or Siglec-15 RNAi. (d) Western blot analysis of STAT1 expression and STAT3 expression in ARO cell transfection with vector-expressing STAT1 or STAT3. Clone formation analysis and flow cytometry analysis of cell proliferation (e) and cell apoptosis (f) in ARO cell transfection with vector-expressing STAT1 and STAT3 and Siglec-15 RNAi. (g) Western blot analysis of cleaved caspase-3 expression and VEGF expression in ARO cell transfection with vector-expressing STAT1 and STAT3 and Siglec-15 RNAi. ^∗∗^*p* < 0.01, ^##^*p* < 0.01, and ^^^^*p* < 0.01 indicated statistical significance compared with control group, scramble group, or STAT3 group.

In this study, THCA cells treated with agonistic anti-CD40 mAb were enrolled into the positive control group. As a result, treatment with 5C11 remarkably suppressed VEGF, STAT1, and STAT3 levels and increased cleaved caspase-3 expression (Figures 6(a)- 6(c)). Meanwhile, Siglec-15 silencing also showed a stronger ability to suppress VEGF, STAT1, and STAT3 levels and increased cleaved caspase-3 expression.

Then, the vectors expressing STAT1 and STAT3 were transfected into THCA cells to upregulate STAT1 and STAT3 expressions in ARO cells (Figure 6(d)). As a result, overexpression of STAT1 and STAT3 promoted cell proliferation and inhibited their apoptosis; however, siglec-15 silencing reversed the above effects (Figures 6(e) and 6(f)). In addition, overexpression of STAT1 and STAT3 inhibited cleaved caspase-3 expression and promoted VEGF expression, which were reversed by siglec-15 silencing (Figure 6(g)).

### 3.7. Siglec-15 Silencing Inhibits Tumor Growth and Strengthens Immune Response by Inhibiting the Activation of STAT1/STAT3 Signaling Pathway

**Figure 7:**
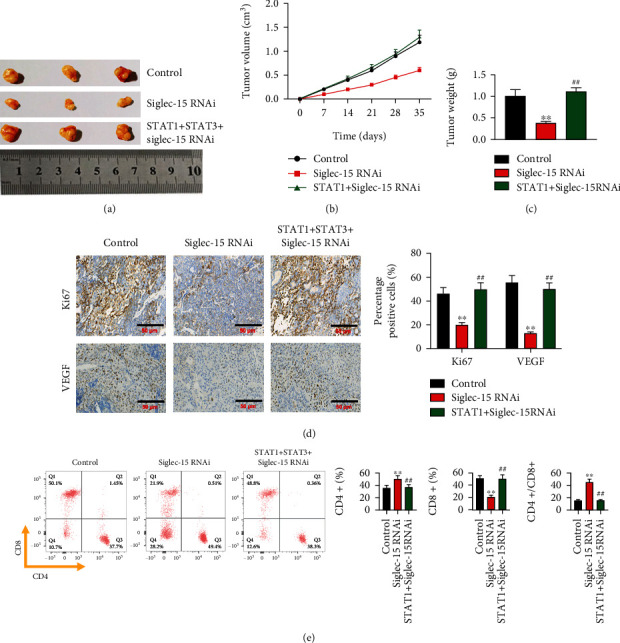
Siglec-15 silencing inhibits tumor growth and strengthen immune via inhibiting of activation of STAT1/STAT3 signaling pathway. (a) Representative image of subcutaneous tumors isolated from nude mice at the experimental endpoint. Quantitative analysis of tumor volumes (b) and tumor weight (c) of the indicated cell xenografts at the indicated time points. (d) Tumor tissue sections from the indicated cell xenografts were prepared and proceeded for H&E staining and anti-Ki-67 and anti-VEGF immunostaining. (e) flow cytometry analysis of the ratio CD4^+^/CD8^+^. ^∗∗^*p* < 0.01 and ^##^*p* < 0.01 indicated statistical significance compared with control group or Siglec-15 RNAi group.

Siglec-15 silencing significantly inhibited the tumorigenicity of THCA cells; however, cotransfection with STAT1 overexpression, STAT3 overexpression, and siglec-15 silencing vectors made no difference to the tumorigenicity of THCA cells (Figures 7(a)–7(c)). This showed that the tumor suppression ability of siglec-15 silencing was counteracted by STAT1 overexpression and STAT3 overexpression. Likewise, siglec-15 silencing reduced VEGF expression, and this impact was reversed by STAT1 overexpression and STAT3 overexpression (Figure 7(d)). Furthermore, siglec-15 silencing increased the CD4^+^/CD8+ ratio. Not surprisingly, STAT1 overexpression and STAT3 overexpression inhibited the regulatory effects of siglec-15 silencing on CD4^+^/CD8^+^ ratio (Figure 7(e)).

## 4. Discussion

Siglec-15 belongs to Siglec family, but it is different from additional family members upon phylogenetic analysis [22]. The extracellular domain of Siglec-15 contains type 2 constant domain (IgC2) and immunoglobulin variable domain (IGV), and it is highly homologous to B7-H1 as well as additional B7 family members in terms of domain composition [23]. Typically, it is reported to be more than 30% homologous to the B7 family, suggesting that Siglec-15 is closely related to the B7 family. Similar to B7 family members, Siglec-15 possibly has immunomodulatory activity. B7-H1 shows mutual exclusion with Siglec-15 within human lung cancer (LC) samples. A study published in the Nature Medicine finds that Siglec-15 serves as an appealing cell surface target in tumor immunotherapy [15]. Firstly, Siglec-15 is lowly expressed within healthy tissues, and its physiology in Siglec-15-deficient mice does not fluctuate at all, suggesting that Siglec-15 may not be the essential molecule for organ and tissue development and survival, and this offers the safe boundary for Siglec-15-blocking treatment. Secondly, Siglec-15 is upregulated in macrophages and tumor cells, rather than healthy tissues, indicating its restricted activity within TME. This makes it possible for Siglec-15 to be the specific tumor-selective antibody for cancer treatment. Thirdly, according to Siglec-15-deficient mouse model study, Siglec-15 shows high immunosuppression on T cell responses at the tumor sites. Finally, in multiple tumor models, Siglec-15-specific mAb reverses T cell inhibition, promotes tumor immunity, and suppresses tumor growth.

In this study, bioinformatics analysis also identified Siglec-15 as an IRG that was highly expressed in THCA tumor samples. Our results further confirmed that Siglec-15 was generally highly expressed in thyroid adenoma, FTC, ATC and PTC, which was significantly associated with poor patient outcomes. As revealed by in vitro functional assays, Siglec-15 knockdown remarkably suppressed THCA cell growth and promoted their apoptosis. These results demonstrate that Siglec-15 is an oncogene for THCA. Combined with KEGG enrichment analysis, Siglec-15 was sensitive to STAT signaling pathways. PD-L1 is recognized as an antitumor immunosuppressor, which reduces PD-L1 expression after siRNA knockdown of STAT1 or STAT3 [24]. These results are confirmed in this study. CD40 activation can activate the anti-PD-1 response [25]. Thus, in this study, treatment with CD40-activated antibodies inhibited THCA cell proliferation, apoptosis, and STAT1 and STAT3 expression. Siglec-15 RNAi also inhibited the expression of STAT1, STAT3, VEGF (a tumor growth factor), and caspase-3 (a pro-apoptosis-related protein) [26]. Further experiments indicated that STAT1 and STAT3 contributed to tumor abrogation by Siglec-15 RNAi. Collectively, these data suggest that Siglec-15 promotes tumor progression and the activation of STAT1/STAT3 signaling pathway, which is associated with tumor immunity.

Tumorigenesis is necessarily accompanied by tumor immunity, in which immunocytes have a critical effect on regulating immunity through the infiltration into TME. CD4^+^ helper T cells and CD8^+^ cytotoxic T cells are in close contact with tumor cells, which have critical effects on tumor immunity [27]. CD4^+^ T cells are antigen-presenting cells (APCs) that coordinate the differentiation of B cells into plasma cells to produce antibodies and activate CD8^+^ T cells. CD8^+^ T cells not only enhance immune response by secreting cytokines but also directly kill tumor cells. CD4^+^ T cells and CD8^+^ T cells together constitute the central hubs of immunomodulation, and their balance plays a critical role in maintaining the body's normal immunity [28]. The decrease in CD4^+^/CD8^+^ ratio suggests suppressed immune levels and the susceptibility to tumor metastasis. The increased immunosuppression degree within TME indicates the stronger neovascularization capacity [29]. In vivo studies showed that Siglec-15 RNAi inhibited tumor growth and increased the CD4^+^/CD8^+^ ratio; however, this was offset by the overexpression of STAT1 and STAT3. This suggests that Siglec-15 plays an immunosuppressive role by activating the STAT1/STAT3 signaling pathway.

Collectively, this work detects the significant upregulation of Siglec-15 within adenoma, ATC, PTC, and FTC tissues. Siglec-15 is the possible oncogene that activates the STAT1/STAT3 signaling pathway to promote THCA cell growth, leading to an increase in the immunosuppression. Therefore, Siglec-15 may be a new immune checkpoint in THCA.

## Figures and Tables

**Table 1:** Correlation between Siglec-15 expression and clinicopathological characteristics in THCA patients.

Characteristics	Number	Siglec-15 expression		*χ* ^2^	*p* values
		Low (*n* = 42)	High (*n* = 44)		
Type					
FTC	16	7	9	15.885	0.001^∗^
PTC	26	21	5		
Adenoma	13	4	9		
ATC	31	10	21		
Gender					
Male	38	19	19	0.037	0.848
Female	48	23	25		
Age (years)					
≥65	37	20	17	0.707	0.400
<65	49	22	27		
Tumor size (cm)					
≤2	43	26	17	4.654	0.031^∗^
>2	43	16	27		
Lymph nodes metastasis					
No	60	28	32	0.374	0.541
Yes	26	14	12		
Distant metastasis					
No	42	29	13	13.420	<0.001^∗^
Yes	44	13	31		

Note: THCA: thyroid cancer; FTC: follicular thyroid carcinoma; PTC: papillary thyroid carcinoma; ATC: anaplastic thyroid cancer. ^∗^*p* < 0.05.

**Table 2:** The multivariate Cox regression analysis between 23 markers and OS in THCA.

	HR	95% CI	*p* value
HSPA1B	3.929	1.746-8.839	0.001
CXCL5	2.813	0.910-8.695	0.073
SIGLEC15	6.559	0.722-59.565	0.095
COLEC12	0.201	0.045-0.889	0.034
AZU1	7.732	1.995-29.961	0.003
APOD	2.232	1.099-4.533	0.026
NFATC2	3.250	1.035-10.206	0.044
SEMA6B	8.843	2.371-32.983	0.001
AGRP	9.330	2.217-39.260	0.002
BMP8A	2.086	1.12-3.885	0.020
IL20RA	42.522	4.641-38.553	0.001
TGFBR3	3.441	0.675-17.531	0.137
LRRK2	1.488	0.864-2.563	0.152
CDKN2A	3.596	1.404-9.209	0.008
GLI1	0.020	0.001-3.670	0.141

Notes: THCA: thyroid cancer; OS: overall survival; HR: hazard ratio; CI: confidence intervals. ^∗^*p* < 0.05.

## Data Availability

The datasets generated/analyzed during the current study are available upon reasonable requests.

## Abbreviations

THCA: Thyroid cancer
PTC: Papillary thyroid carcinoma
FTC: Follicular thyroid carcinoma
ATC: Anaplastic thyroid cancer
TCIGs: THCA-related immune genes
BPs: Biological processes
CCs: Cellular components
MFs: Molecular functions
TCAA: T cell activity array system
DEGs: Differentially expressed genes
WHO: World Health Organization
PBS: Phosphate-buffered saline
FITC: Fluorescein isothiocyanate.
